# Pharmacological Overview of the BGP-15 Chemical Agent as a New Drug Candidate for the Treatment of Symptoms of Metabolic Syndrome

**DOI:** 10.3390/molecules25020429

**Published:** 2020-01-20

**Authors:** Ágota Pető, Dóra Kósa, Pálma Fehér, Zoltán Ujhelyi, Dávid Sinka, Miklós Vecsernyés, Zoltán Szilvássy, Béla Juhász, Zoltán Csanádi, László Vígh, Ildikó Bácskay

**Affiliations:** 1 Department of Pharmaceutical Technology, Faculty of Pharmacy, University of Debrecen, Egyetem tér 1, H-4032 Debrecen, Hungary; peto.agota@pharm.unideb.hu (Á.P.); kosa.dora@pharm.unideb.hu (D.K.); feher.palma@pharm.unideb.hu (P.F.); ujhelyi.zoltan@pharm.unideb.hu (Z.U.); sinka.david@pharm.unideb.hu (D.S.); vecsernyes.miklos@pharm.unideb.hu (M.V.); 2 Department of Pharmacology and Pharmacotherapy, Faculty of Medicine, University of Debrecen, Nagyerdei Körút 98, H-4032 Debrecen, Hungary; szilvassy.zoltan@med.unideb.hu (Z.S.); juhasz.bela@med.unideb.hu (B.J.); 3 Institute of Cardiology, University of Debrecen, Móricz Zsigmond Körút 22, H-4032 Debrecen, Hungary; csanadi.zoltan@med.unideb.hu; 4 Biological Research Center, Hungarian Academy of Sciences, Temesvári Körút 62, H-6726 Szeged, Hungary; vigh@brc.hu

**Keywords:** BGP-15, chaperone co-inducer, PARP inhibitor, insulin sensitizer

## Abstract

BGP-15 is a new insulin sensitizer drug candidate, which was developed by Hungarian researchers. In recent years, numerous research groups have studied its beneficial effects. It is effective in the treatment of insulin resistance and it has protective effects in Duchenne muscular dystrophy, diastolic dysfunction, tachycardia, heart failure, and atrial fibrillation, and it can alleviate cardiotoxicity. BGP-15 exhibits chemoprotective properties in different cytostatic therapies, and has also proven to be photoprotective. It can additionally have advantageous effects in mitochondrial-stress-related diseases. Although the precise mechanism of the effect is still unknown to us, we know that the molecule is a PARP inhibitor, chaperone co-inducer, reduces ROS production, and is able to remodel the organization of cholesterol-rich membrane domains. In the following review, our aim was to summarize the investigated molecular mechanisms and pharmacological effects of this potential API. The main objective was to present the wide pharmacological potentials of this chemical agent.

## 1. Introduction

BGP-15 was originally developed to treat insulin resistance by N-Gene Research Laboratories Inc., however, many additional pharmaceutical effects have been revealed in the last few decades [1]. The BGP-15 compound was discovered while investigating heat shock proteins, which are essential in the functioning of the immune system. This pharmacon seemed to be a promising therapeutic agent, with several beneficial effects based on these experiments. When it was discovered, it was thought that the molecule may be able to treat many diseases. The compound targets a well-known mechanism of the human body; the stimulation of heat shock protein expression, which leads to an increased stress response and thus increased energy supply in the cells [2]. BGP-15 is a versatile compound, and many research groups are paying significant attention to the molecule and investigating its effects all around the world. It has been reported to be safe and well tolerated [3]. It entered into clinical phase II trials for the indication of insulin resistance. [4]. The objective of this review is to summarize BGP-15’s effects, and the experiments that have been performed to increase the knowledge about this active pharmaceutical ingredient (API).

## 2. Chemical Properties

**Figure 1:**
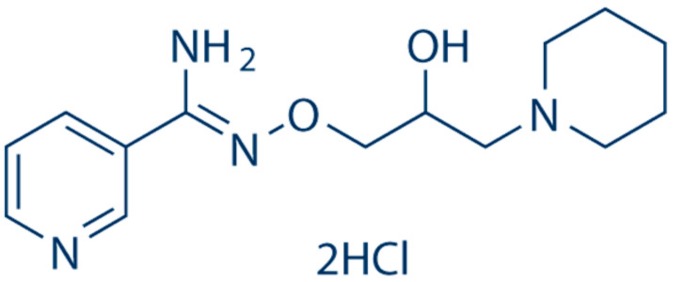
Chemical structure of BGP-15 [5].

BGP-15 (C_14_H_22_N_4_O_2_·2HCl) is a nicotinic amidoxime derivate (Figure 1), (Z) (*N*′-(2-hydroxy-3-(piperidin-1-yl)propoxy)-3-pyridine-carboximidamide), and a solid material. Its molecular weight is 351,272 g/mol. Its solubility in deionized water is 28 mg/mL at 25 °C [6,7]; BGP-15 is a small molecule and its water solubility is good. Water solubility and molecular weight are always crucial points in the case of formulation of a certain API. Based on these pieces of information, working with this chemical agent—from a pharmaceutical technology aspect—is not problematic at all.

## 3. Mechanisms of Effects

**Figure 2:**
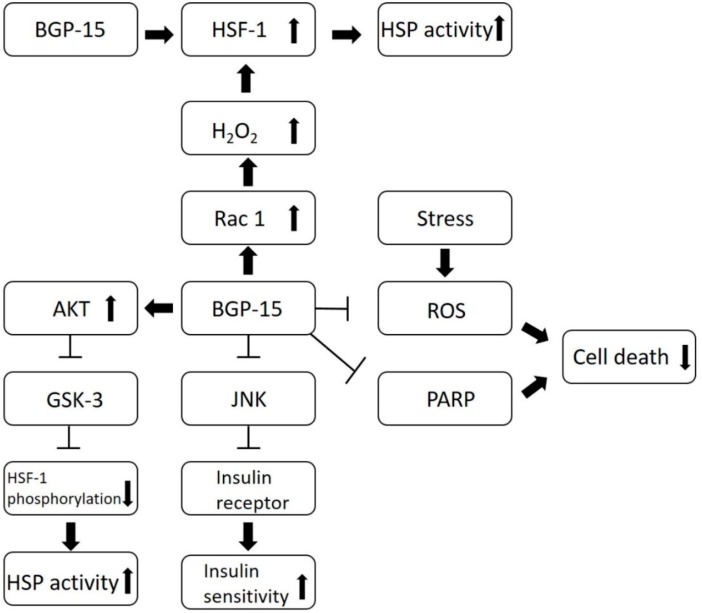
Mechanisms of effects of BGP-15.

Several research groups are currently studying this molecule; however, the exact mechanisms of its effects are still unknown to us. All of the beneficial effects of BGP-15 are based on or linked to the following mechanisms (Figure 2):

- BGP-15 inhibits the acetylation of heat shock factor 1 (HSF-1), thus increasing heat shock protein (HSP) induction. It is a co-inducer of Hsp72 [3].
- BGP-15 blocks JNK, an inflammatory cytokine, thus preventing JNK from inhibiting the phosphorylation of the insulin receptor, which results in increased insulin sensitivity [8,9,10].
- BGP-15 is a poly (adenosine 5′-diphosphate)–ribose] polymerase 1 (PARP-1) inhibitor, and is able to reduce mitochondrial ROS production as well. Pharmacological inhibition of PARP and reducing the production of reactive oxygen species (ROS) can be effective in a wide selection of diseases, by protecting the cells against death [11].
- BGP-15 increases AKT levels moderately, and AKT is a signaling factor in the insulin-signaling pathway. This results in increased glucose uptake and the survival of the cells. AKT activation causes the deactivation of GSK-3, thus the kinase inhibitory phosphorylation of HSF-1 will decrease [8,9].
- BGP-15 activates Rac1, a signaling protein, which increases the level of H_2_O_2_. H_2_O_2_ acts as a messenger and increases HSF-1, thus promoting HSP induction [8,9].
- BGP-15 is able to remodel lipid rafts and to increase membrane fluidity, which is important because stiff membranes are limiting the cellular stress response [8,9,12,13].

BGP-15 inhibits JNK’s inhibitory effect on the insulin receptor, thus insulin sensitivity is improved. BGP-15 blocks the PARP enzyme, which decreases cell death. BGP-15 is able to inhibit mitochondrial ROS production, which also results better survival of cells. Moreover, BGP-15 increases AKT phosphorylation, leading to GSK-3 deactivation, thus the inhibitory phosphorylation of HSF-1 will decrease and HSP induction will increase. It also inhibits the acetylation of HSF-1, which results in increased HSP activity. BGP-15 activates Rac 1, thus the H_2_O_2_ level will increase, which will activate HSF-1 and induce HSP activity.

## 4. Pharmacology

BGP-15 has entered clinical trials in insulin resistance indication, but its effectiveness has previously been demonstrated in various other diseases as well. The main indication for this compound is the treatment of diabetes and insulin resistance [3,14]. It might also be an adjuvant in the therapy of Duchenne muscular dystrophy (DMD) [15]. Moreover, BGP-15 can be a cardioprotective agent in ischemia-reperfusion injury treatment [16], it improves diastolic dysfunction in diabetic cardiomyopathy [17], and it can be effective in treating heart failure as well [18]. In addition, it is proven to be neuroprotective in cisplatin or taxol-induced peripheral neuropathy [19], and is also a promising photoprotective agent [20]. It additionally has protective effects in nephrotoxicity induced by cisplatin [21]. Administration of BGP-15 also has potential in traumatic brain injury [22], and it could be effective in the therapy of ventilation induced diaphragm dysfunction [23]. This molecule can be efficient in oxidative-stress-related diseases as well [24].

As mentioned above, BGP-15 has various beneficial effects, but we know the most about its insulin sensitizing effect. Some of the other beneficial effects require further investigation to expand our knowledge and learn more about the API. There are many ongoing experiments, so other effects and data are expected to be published in the future regarding BGP-15.

## 5. Preclinical Studies

In drug development, preclinical studies must be performed to estimate human responses and extend the knowledge regarding the API before clinical studies can begin. They are usually performed with the help of in vitro and in vivo tests [25]. In the series of investigations related to BGP-15, research groups have selected different types of cell culture models and animal models, which are summarized in this chapter.

### 5.1. Cell Culture Models

MEF cells (mouse embryonic fibroblasts) were selected to investigate the effects of BGP-15 on the heat shock response [26]. These fibroblasts were harvested from mice embryos and applied for the examination of molecular mechanisms [27]. To determine Hsp72 effectiveness in the treatment of obesity-induced insulin resistance, L6 myotubes were deployed [28]. This cell line is a suitable in vitro model for the investigation of glucose uptake [29]. To evaluate the effect of BGP-15 on mitochondrial fragmentation, WRL-68, C_2_C_12_, A549 and Sf9 cell cultures were maintained [30]. Mitochondrial ROS production has also been studied on the WRL-68 cell line [24]. These HeLa derivatives can be a useful model for the examination of hepatocyte function in vitro [31]. C_2_C_12_ cells are immortalized mouse myoblast cells with the ability to quickly develop into skeletal or cardiac muscle cells [32]. A549 cells are human lung adenocarcinoma cells that are commonly used as a type II pulmonary epithelial cell model [33]. Sf9 are derived from the pupal ovarian tissue of *Spodoptera frugiperda*. This cell line is often used because of its high-level protein expression [34]. Besides the WRL-68 cell line, mitochondrial ROS production has also been studied using H9c2 and U-251 cells [24]. H9c2 cells are rat heart myoblasts, and are often used to examine the metabolic activity of the heart [35]. U-251 MG cells are human malignant glioma cells [36]. B16-F10 mouse melanoma cells were maintained to study the BGP-15 effect mechanism [37]. NRVM are neonatal rat ventricular myocytes originating from Sprague–Dawley strain rats’ ventricles, and were used for the investigation of the therapeutic role of BGP-15 in heart failure and atrial fibrillation [18].

### 5.2. Animal Models

Wistar rats are one of the most commonly used rodents for animal experiments. Several effects of BGP-15 have been investigated using Wistar rats. The insulin sensitizer effect [14], the chemoprotective action in cisplatin or taxol therapy [19], the cardioprotective effect in imatinib therapy [38], and its effect in ischemia-reperfusion injury [11,16] have all been studied with the help of these rats. In a few cases, only some of their organs have been used. For example, the cardioprotective action of the molecule has been investigated with the help of the Langendorff heart perfusion system [11,16,38]. This is a suitable and versatile in vitro model for different experiments carried out on smaller animals’ (rats, mice, and rabbits) hearts [39].

There have been other types of rats used for the investigation of BGP-15. The effect of cardiovascular (CV) complications caused by diabetes and the insulin sensitizing effect of the API have been studied on Goto–Kakizaki rats [17]. These animals are one of the most commonly used rat models of type 2 diabetes mellitus. They display mild hyperglycemia and damaged glucose tolerance, while being normotensive and non-obese [17]. Besides the insulin-sensitizing effect, the role of BGP-15 in alleviating ventilation-induced diaphragm dysfunction has been examined on Sprague–Dawley rats [40]. The advantage of these rats is their calmness and easy handling [41]. On Zucker obese rats, the insulin sensitivity of BGP-15 has been investigated in combination with rimonabant [4]. These types of rats are a suitable genetic model for studying obesity and hypertension. They are insulin resistant, but not hyperglycemic [42].

The other commonly used small animals are mice. Several types of mice have been selected for experiments relating to BGP-15.

The protective effect of BGP-15 in liver injury caused by acetaminophen has been investigated on CD-1 mice [1]. These mice are a general model for safety and efficacy testing [43]. To test the role of Hsp72 in the treatment of obesity-induced insulin resistance, leptin deficient (ob/ob) mice were selected [44]. They are a suitable model of mild type 2 diabetes and obesity [45]. The efficacy of BGP-15 in the therapy of Duchenne muscular dystrophy was tested on mdx and dko mice [15,46]. Although mdx mice (X-chromosome-linked DMD) have no functional dystrophin, unfortunately the DMD phenotype is mild in comparison to the human disease. The dko (dystrophin–utrophin double knockout) mice lack both utrophin and dystrophin, and this loss results in a much more severe phenotype [47]. For the investigation of the photoprotective effect of the pharmacon, hairless mice were used (VAF/plus CRL: hr/hr BR); they have the obvious advantages of topical administration being much easier, and the results being more visible and easier to detect [20].

The beneficial effect of BGP-15 has been investigated in PCOS, as an ER stress inhibitor [48], in oxaliplatin therapy induced skeletal myopathy and ROS production [49], and in oxaliplatin induced intestinal dysfunction [50]. The investigations were carried out on BALB/c mice. They are useful in experiments for both immunology and cancer [51].

To study the protective effect of the pharmacon in cisplatin caused nephrotoxicity, NMRI CV1 mice and BD2F1 mice were selected [21]. NMRI CV1 mice are models for toxicology, teratology, physiology, and pharmacology [52]. BD2F1 mice bear syngeneic lymphoid tumors [51].

Hsp110 deficient mice have been used to investigate whether the induction of Hsp70/Hsp110 can protect mice from traumatic brain injury [22].

The effect of BGP-15 on the contractile function and morphology of regenerating soleus muscles was examined on CB6F1 mice [53]. These animals are ideal for transplantation research or monoclonal antibody production [54].

For the assessment of the therapeutic potential of BGP-15 in heart failure (HF) and atrial fibrillation (AF), two mouse models were used. Both develop HF over time and exhibit an increased susceptibility to AF; they are the cardiac-specific dnPI3K-Mst1 Tg mice20 and the cardiac-specific MURC Tg mice40 [18].

The effect of BGP-15 on mitochondrial dysfunction in obese mothers’ oocytes was evaluated. For this purpose, a mouse model of Alstrom syndrome was selected. This is a rare genetic disease which causes hyperphagia, leading to obesity, hyperinsulinemia, and diabetes [55]. To study the effect of BGP-15 in gestational diabetes, mellitus female C57BL/6 mice were involved in the study and mated with non-diabetic male mice to cause pregnancy. Brown adipose tissue was removed at the end of the 15th week [56].

For investigation of the insulin sensitizer effect of BGP-15, white New Zealand rabbits were selected. Their main advantage is their quick growth rate [14].

For the assessment of the role of BGP-15 in the treatment of tachycardia, Drosophila melanogaster was selected. These fruit flies have a pumping heart and 85% of their genes have human homologues, and many of these genes are associated with human cardiac diseases such as heart failure, arrhythmias, and cardiomyopathy [57]. All animal models and the outcome of the related experiments are summarized in Table 1.

## 6. Potential Effects

### 6.1. Cardiovascular Effects

BGP-15 shows several beneficial cardiovascular effects, and has been investigated and studied in a wide range of pathological conditions in several disease models (Table 2). It has proven to be a promising therapeutic cardioprotective agent in ischemic heart disease [11]. Szabados E. et al. investigated the molecular mechanism by which BGP-15 can protect the heart from ischemia–reperfusion injury in a Langendorff heart perfusion system. They found that PARP is an important target of BGP-15, and BGP-15 decreases ROS levels and cell injury during ischemia–reperfusion [11].

Bombicz M. et al. investigated the effectiveness of BGP-15 in relieving cardiac dysfunction on Goto–Kakizaki rats, and found that BGP-15 is able to repair diastolic dysfunction in diabetic cardiomyopathy [17]. Sapra G. et al. evaluated the therapeutic potential of BGP-15 in a mouse model which can develop heart failure and is susceptible to atrial fibrillation. They came to the conclusion that it ameliorates cardiac function and reduces arrhythmic episodes for those who suffer from heart failure and atrial fibrillation [18]. Zhang D. et al. treated Drosophila melanogaster with BGP-15 in tachycardia indication, and according to their experiments, BGP-15 was able to protect the heart against tachycardia [57].

### 6.2. The Effect of BGP-15 in Duchenne Muscular Dystrophy

BGP-15 has potential in Duchenne muscular dystrophy (DMD); it could be used as a beneficial adjuvant to therapy since it is able to improve the condition of cardiac pathology, and it ameliorates dystrophic pathology (Table 3) [15,46]. Gehrig S. M. et al. tested whether overexpression of Hsp72 in dystrophic muscles would ameliorate SERCA function. They found that treatment of dystrophic mice with BGP-15, a pharmacological co-inducer of HSP 72, improved the pathology of DMD and extended the lifetime [15]. Kennedy T. L. et al. investigated whether treatment with BGP-15 was beneficial in older mdx and dko mice when advanced pathology had already occurred. Based on the results of their experiments, it appears to only be effective in the early stage of the disease. Thus, it can be concluded that BGP-15 has a therapeutic window [46].

### 6.3. Chemo and Cytoprotective Effects of BGP-15

It has been proven that the pharmacon can protect myenteric neurons of Balb/c mice from oxaliplatin, making it a useful addition in chemotherapy; it might relieve some of the serious side-effects of cytostatics. McQuade R. M. et al. tested whether BGP-15 is able to alleviate intestinal dysfunction and oxaliplatin-induced enteric neuropathy. They came to the conclusion that BGP-15 is able to improve oxidative stress and increase enteric neuronal survival. It alleviated oxaliplatin-induced intestinal dysfunction [50]. Racz I. et al. investigated the effects of BGP-15 on antitumor activity and nephrotoxicity of cisplatin. They proved that BGP-15 treatment prevented the development of acute renal failure in NMRI CV1 mice caused by cisplatin treatment [21].

Sarszegi Zs. et al. tested how BGP-15 would affect imatinib-caused cardiotoxicity. They discovered that the oxidative damage in the heart was reversible by administration of BGP-15 [38]. According to Bárdos G. et al., BGP-15 is a promising drug that can reduce toxic side effects of taxol and cisplatin [15]. This beneficial effect of BGP-15 does not compromise the antitumor activity of the cytostatics [19,21,38,50]. The relevant experiments are summarized in Table 4.

### 6.4. BGP-15’s Effect in Liver Injury

Nagy G. et al. investigated the effects of BGP-15 in acetaminophen-provoked hepatocellular injury (Table 5). BGP-15 is able to attenuate the degree of paracetamol-induced cell death, which could be an important effect considering the severity of the disease [1].

### 6.5. Insulin Sensitizing Effect of BGP-15

The main indication of the molecule is that it is able to increase insulin sensitivity in an insulin resistant state; moreover, it improves the insulin resistance caused by atypical antipsychotic drugs (AAPD). Furthermore, it is able to prevent the metabolic side effects caused by AAPDs, and also potentiates the insulin sensitizing effect of rimonabant (Table 6) [3,4,14,58,59,60].

Literáti-Nagy B. et al. tried to quantify and demonstrate the insulin sensitizing effect of BGP-15. They found that both the 200 and 400 mg BGP-15 dose groups showed markedly increased insulin sensitivity [3]. In another study Literáti-Nagy B. et al. investigated the effect of BGP-15 in the treatment of olanzapine-caused metabolic side effects. According to the results, BGP-15 markedly decreased olanzapine-induced insulin resistance [58]. In a later experiment, Literáti-Nagy B. et al. evaluated the insulin sensitizing effect of BGP-15 alone, and compared it with metformin, rosiglitazone, and glibenclamide. As an insulin sensitizer, BGP-15 produced better results than metformin and equaled the effect of rosiglitazone. It also proved to be efficient in increasing insulin sensitivity in combination with a sulfonylurea agent (glibenclamide). Treatment with different doses of BGP-15 showed the insulin sensitizing effect in cholesterol-fed rabbits, but not in normal rabbits. The most effective dose was 20 mg/kg. These experiments confirmed that BGP-15 has a beneficial effect only in the insulin-resistant state [14]. Literáti-Nagy Zs. et al. tested the insulin sensitizing effect of BGP-15 in combination with rimonabant. They found that BGP-15 potentiates the insulin sensitizing effect of rimonabant; it occurs at much lower doses than would be expected if the two drugs were administered alone [4].

### 6.6. Effects of BGP-15 in Skin Injury, TBI, and VIDD

Farkas B. et al. investigated the protective effect of BGP-15 against injuries caused by UV rays. BGP-15 treatment markedly decreased the number of sunburn cells in UV radiation exposed skin; BGP-15 has a DNA protective effect if applied topically [20]. Eroglu B. et al. examined whether those drugs that increase Hsp70/Hsp110 levels are able to protect cells against traumatic brain injury (TBI). Mice were subjected to TBI and then BGP-15 was applied. Hsp70/Hsp110 have a significant role in neuronal survival after TBI, and the inducers of these heat shock proteins have beneficial effects in the reduction of the pathological consequences of TBI, thus BGP-15 has potential in TBI as it helps promote the survival of neurons [22]. Salah H. et al. assessed the therapeutic effects of BGP-15 on ventilation-induced diaphragm dysfunction (VIDD). BGP-15 increased diaphragm muscle fiber force generation capacity, thus decreasing the negative effects of mechanical ventilation on diaphragm muscle function [23]. The relevant experiments are summarized in Table 7.

### 6.7. Gynecological Diseases and ROS-Related and Inflammatory Diseases

Recently, it has been discovered that BGP-15 might be useful in gynecological diseases (Table 8). Takahashi N. et al. investigated the effects of BGP-15 in the treatment of PCOS mice. BGP-15 treatment reduced interstitial fibrosis and collagen deposition in the ovaries [48]. Xing B. et al. evaluated the changes in glucose and lipid metabolism levels after activation of Hsp70 with BGP-15. According to the results, BGP-15 was able to significantly reduce abnormal post-natal weight gain [56]. 

Sumegi K. et al. tested whether the protective effects of BGP-15 arise in connection with reducing mitochondrial ROS production and through preserving mitochondrial integrity. According to their results, BGP-15 exhibits protective effects against the progression of ROS-related and inflammatory diseases by decreasing mitochondrial ROS production [24].

More details about the experiments mentioned in the chapter “Potential Effects” can be found in the Supplementary Materials.

## 7. Aspects of Pharmaceutical Technology

BGP-15 is a versatile agent with many potential applications, and it can be administered both internally and externally. Topical use has already been investigated, and in the future different types of semisolid dosage forms could be developed and formulated to utilize its photoprotective effect. Since it is a promising insulin sensitizer agent, it could revolutionize the current therapy for insulin resistance and type 2 diabetes. This is a suitable pharmacon for an orally administered solid dosage form. The cardioprotective effect is also promising and, besides the conventional tablets and capsules, transdermal patches could be an interesting and useful addition to formulations of this pharmacon, which could improve patient compliance.

## 8. Clinical Trials of BGP-15

BGP-15 is a novel drug candidate, and it is currently in phase II human clinical trials. Based on the previous clinical trial entitled “Safety and Efficacy of BGP-15 in Patients With Type 2 Diabetes Mellitus” sponsored by N-Gene Research Laboratories Inc., BGP-15 has proven to be effective and well tolerated in insulin resistance and Type 2 Diabetes Mellitus, however, it cannot not currently available on the market yet. Unfortunately, the main indication (Type 2 Diabetes Mellitus) needs to be revaluated, since it has not been verified. Careful controlling of biostatistical calculation detected that the patient number in the groups should be increased. Therefore, a clinical trial with greater patient numbers with repeated biostatistical calculations should be designed and carried out in the future. Nevertheless, new promising preclinical results emerged, and the efficacy of BGP-15 as a chaperone co-inducer was reported in early contractile dysfunction [61]. Based on this article, a new clinical trial is ready to be submitted regarding the indication of inappropriate sinus tachycardia, which, hopefully, will be successful due to the appropriate design and the suitable number of patients [62]. 

## 9. Summary

BGP-15 is a promising and versatile molecule with many beneficial effects that have been discovered over the last decade [3,4]. It exhibits insulin sensitizing properties [3,4,14,58,59,60], and protective effects in Duchenne muscular dystrophy [15,46], diastolic dysfunction, tachycardia, heart failure, and atrial fibrillation, and it can fend off cardiotoxic effects [11,17,18,57]. It has proven to be chemoprotective in different cytostatic therapies, and photoprotective as well [19,20,21,38,50]. It can also be effective in mitochondrial-stress-related diseases [24]. Although the exact mechanism of action has not been elucidated, it has been certified that the molecule is a chaperone co-inducer, PARP inhibitor, reduces ROS production, and it is able to increase membrane fluidity [3,8,9,10,11]. These modes of action may explain why the pharmacon has so many beneficial effects. The effects and the connected therapeutic possibilities are currently being investigated by many research groups all over the world.

If BGP-15 successfully passes clinical trials in the future, it could be therapeutically effective for the treatment of many diseases. In this review, many potential pharmacological effects of BGP-15 were presented. Based on these pharmacological profiles, it could be registered as an original medicine; however, the determination of a specific indication field is needed. Further human clinical trials might certify the effective role of BGP-15 in a different medical treatment.

## Figures and Tables

**Table 1:** Summary of the studied effects and diseases, with the help of animal models.

Animal Model	Studied Effect or Disease
Wistar rats [11,14,16,19]	insulin sensitizing effect, chemoprotective action, cardioprotective effect
Goto-Kakizaki rats [17]	insulin sensitizing effect, diabetes caused CV complications
Sprague–Dawley rats [40]	insulin sensitizing effect, the role of BGP-15 in alleviating ventilation-induced diaphragm dysfunction
Zucker obese rats [4]	insulin sensitizing effect in combination of rimonabant
CD-1 mice [1]	liver injury
Leptin deficient (ob/ob) mice [44]	testing the role of Hsp72 effective in the treatment of obesity-induced insulin resistance
mdx and dko mice [15,46]	Duchenne muscular dystrophy
hairless mice (VAF/plus CRL: hr/hr BR) [20]	photoprotective effect
BALB/c mice [48,49,50]	PCOS, oxaliplatin therapy induced skeletal myopathy and intestinal dysfunction
NMRI CV1 mice and BD2F1 mice [21]	cisplatin caused nephrotoxicity
Hsp110 deficient mice [22]	traumatic brain injury
CB6F1 mice [53]	effect of BGP-15 on the contractile function and morphology of regenerating soleus muscles
cardiac-specific dnPI3K-Mst1 Tg mice20 and cardiac-specific MURC Tg mice40 [18]	heart failure
mouse model of Alstrom syndrome [55]	mitochondrial dysfunction
C57BL/6 mice [56]	gestational diabetes mellitus
white New Zealand rabbits [14]	insulin sensitizing effect
Drosophila melanogaster [57]	tachycardia

**Table 2:** Summary of investigations related to the cardiovascular effects of BGP-15.

Main Effect	Dose
Reconstruction of diastolic dysfunction [17]	10 mg/kg BGP-15, per os
	100 mg/kg metformin, per os
Amelioration of cardiac function and reduction of arrhythmic episodes [18]	15 mg/kg BGP-15
	50 µM BGP-15 on the cells
Endogenous HSP overexpression and protection against tachycardia remodeling [57]	1 mM BGP-15
Reduction of ROS levels and cell injury during ischemia–reperfusion [11]	The medium contained 40 mg/L of BFP-15

**Table 3:** Summary of the investigations related to the of BGP-15 in the therapy of DMD.

Main Effect	Dose
Improvement of DMD pathology and extension of lifetime [15]	15 mg/kg per day, oral gavage
Improvement of cardiac pathology in DMD, but skeletal muscle function was not improved in older mdx or dko mice [46]	15 mg/kg BGP-15

**Table 4:** Summary of the performed investigations related to the aforementioned effects of BGP-15.

Main Effect	Dose
Reduction of the toxic side effects of taxol and cisplatin without compromising their antitumor effect [19]	50, 100, 200 mg/kg of BGP-15, per os, once daily throughout the experiment
	1.5 mg/kg cisplatin, intraperitoneally, once daily, for 5 days
	5 mg/kg taxol, intraperitoneally, every other day for 10 days
Reversal of oxidative damage in the heart, caused by imatinib [38]	200 µM BGP-15
Prevention of the development of acute renal failure caused by cisplatin treatment [21]	100, 200 mg/kg BGP-15 shortly before cisplatin treatment
Alleviation of oxaliplatin-induced intestinal dysfunction, which eased the gastrointestinal side-effects of chemotherapy [50]	15 mg/kg BGP-15
	3 mg/kg oxaliplatin

**Table 5:** Summary of the investigation related to the effect of BGP-15 in paracetamol-induced cell death.

Main Effect	Dose
Prevention of translocation of AIF (apoptosis inducing factor) and mitochondrial depolarization. [1]	10, 20, 100, 200 mg/kg BGP-15
	450 mg/kg acetaminophen

**Table 6:** Summary of the investigations related to the insulin sensitizing effect of BGP-15.

Main Effect	Dose
Reduction of olanzapine-induced insulin resistance [58]	400 mg of BGP-15 or placebo for 17 days
	5 mg of olanzapine for 3 days and 10 mg for 14 days
Increase of insulin sensitivity [3]	200 mg or 400 mg of BGP-15, or placebo once daily, for 4 weeks
Production of better results than metformin and rosiglitazone Increase of insulin sensitivity in combination with a sulfonylurea agent (glibenclamide) Increase of insulin sensitizing effect in cholesterol-fed rabbits, but not in normal rabbits. [14]	5, 10, 20, 30, or 50 mg/kg of BGP-15 per os 2 mg/kg rosiglitazone per os 100 mg/kg metformin per os 1 mg/kg glibenclamide per os
Intensification of insulin sensitizing effect of rimonabant; [4]	10 mg/kg rimonabant 30 mg/kg rimonabant 3 mg/kg BGP-15 10 mg/kg BGP-15 for 5 days

**Table 7:** Summary of the investigations related to the aforementioned effects of BGP-15.

Main Effect	Dose
Decrease of the number of sunburn cells in UV radiation exposed skin	5–20% concentration of BGP-15 in the cream
DNA protective effect if applied topically [20]	
Beneficial effects in the reduction of the pathological consequences of TBI [22]	15 mg/kg BGP-15, per os
Increasing diaphragm muscle fiber force generation capacity.	40 mg/kg BGP-15 iv.
Decreasing the negative effects of mechanical ventilation on diaphragm muscle function [23]	

**Table 8:** Summary of the investigations related to effects of BGP-15 in gynecological diseases.

Main Effect	Dose
Reduction of interstitial fibrosis and collagen deposition in the ovaries [48]	3 mg/100g of body weight (BGP-15)
Reduction of abnormal weight gain after pregnancy [56]	100 mg/kg BGP-15
Prevention of ROS-related and inflammatory disease progression [24]	50 µM BGP-15

## Supplementary Materials

The following are available online at https://www.mdpi.com/1420-3049/25/2/429/s1. Table S1: Summary of the investigations related to the cardiovascular effects of BGP-15. Table S2: Summary of the investigations related to the effect of BGP-15 in the therapy of DMD. Table S3: Summary of the investigations related to the chemo and cytoprotective effects of BGP-15. Table S4: Summary of the investigation related to the effect of BGP-15 in paracetamol-induced cell death. Table S5: Summary of the investigations related to the insulin sensitizing effect of BGP-15. Table S6: Summary of the investigations related to the effects of BGP-15 in skin injury, TBI, and VIDD. Table S7: Summary of the investigations related to effects of BGP-15 in gynecological diseases.

## References

[1] Nagy G., Szarka A., Lotz G., Dóczi J., Wunderlich L., Kiss A., Jemnitz K., Veres Z., Bánhegyi G., Schaff Z. (2010). BGP-15 inhibits caspase-independent programmed cell death in acetaminophen-induced liver injury. Toxicol. Appl. Pharmacol..

[2] Origo. https://www.origo.hu/egeszseg/20120419-tudomanyos-attorest-hozhat-a-magyar-felfedezes-cukorbetegseg-inzulin.html.

[3] Literáti-Nagy B., Kulcsár E., Literáti-Nagy Z., Buday B., Péterfai É., Horváth T., Tory K., Kolonics A., Fleming A., Mandl J. (2009). Improvement of insulin sensitivity by a novel drug, BGP-15, in insulin-resistant patients: A proof of concept randomized double-blind clinical trial. Horm. Metab. Res..

[4] Literáti-Nagy Z., Tory K., Literáti-Nagy B., Bajza Á., Vígh L., Vígh L., Szilvássy Z. (2013). Synergetic insulin sensitizing effect of rimonabant and BGP-15 in zucker-obes rats. Pathol. Oncol. Res..

[5] Chemical Structure. https://www.selleckchem.com/products/bgp-15.html.

[6] Sigma Aldrich. https://www.sigmaaldrich.com/catalog/product/sigma/b4813?lang=hu&region=HU.

[7] U.S. National Library of Medicine https://pubchem.ncbi.nlm.nih.gov/compound/bgp-15..

[8] Hooper P.L., Balogh G., Rivas E., Kavanagh K., Vigh L. (2014). The importance of the cellular stress response in the pathogenesis and treatment if type 2 diabetes. Cell Stress Chaperones..

[9] Crul T., Toth N., Piotto S., Literati-Nagy P., Tory K., Haldimann P., Kalmar B., Greensmith L., Torok Z., Balogh G. (2013). Hydroximic Acid Derivatives: Pleiotropic Hsp Co-Inducers Restoring Homeostasis and Robustness. Curr. Pharm. Des..

[10] Jeffrey K., López J.C., Schubert C., Stevens K. (2008). Melting away. Nat. Med..

[11] Szabados E., Literati-Nagy P., Farkas B., Sumegi B. (2000). BGP-15, a nicotinic amidoxime derivate protecting heart from ischemia reperfusion injury through modulation of poly(ADP-ribose) polymerase. Biochem. Pharmacol..

[12] Escribá P.V., Busquets X., Inokuchi J., Balogh G., Török Z., Horváth I., Harwood J.L., Vígh L. (2015). Membrane lipid therapy: Modulation of the cell membrane composition and structure as a molecular base for drug discovery and new disease treatment. Prog. Lipid Res..

[13] Gungor B., Vanharanta L., Hölttä-Vuori M., Pirhonen J., Petersen N.H.T., Gramolelli S., Ojala P.M., Kirkegaard T., Ikonen E. (2019). HSP70 induces liver X receptor pathway activation and cholesterol reduction in vitro and in vivo. Mol. Metab..

[14] Literáti-Nagy B., Tory K., Peitl B., Bajza Á., Korányi L., Literáti-Nagy Z., Hooper P.L., Vígh L., Szilvássy Z. (2014). Improvement of Insulin Sensitivity by a Novel Drug Candidate, BGP-15, in Different Animal Studies. Metab. Syndr. Relat. Disord..

[15] Gehrig S.M., van der Poel C., Sayer T.A., Schertzer J.D., Henstridge D.C., Church J.E., Lamon S., Russel A.P., Davies K.E., Febbraio M.A. (2012). Hsp72 preserves muscle function and slows progression of severe muscular dystrophy. Nature.

[16] Halmosi R., Berente Z., Osz E., Toth K., Literati-Nagy P., Sumegi B. (2001). Effect of poly(ADP-ribose) polymerase inhibitors on the ischemia-reperfusion-induced oxidative cell damage and mitochondrial metabolism in Langendorff heart perfusion system. Mol. Pharmacol..

[17] Bombicz M., Priksz D., Gesztelyi R., Kiss R., Hollos N., Varga B., Nemeth J., Toth A., Papp Z., Szilvassy Z. (2019). The Drug Candidate BGP-15 Delays the Onset of Diastolic Dysfunction in the Goto-Kakizaki Rat Model of Diabetic Cardiomyopathy. Molecules.

[18] Sapra G., Tham Y.K., Cemerlang N., Matsumoto A., Kiriazis H., Bernardo B.C., Henstridge D.C., Ooi J.Y.Y., Pretorius L., Boey E.Y.H. (2014). The small-molecule BGP-15 protects against heart failure and atrial fibrillation in mice. Nat. Commun..

[19] Bárdos G., Móricz K., Jaszlits L., Rabloczky G., Tory K., Rácz I., Bernáth S., Sümegi B., Farkas B., Literáti-Nagy B. (2003). BGP-15, a hydroximic acid derivative, protects against cisplatin- or taxol-induced peripheral neuropathy in rats. Toxicol. Appl. Pharmacol..

[20] Farkas B., Magyarlaki M., Csete B., Nemeth J., Rabloczky G., Bernath S., Literáti-Nagy P., Sümegi B. (2002). Reduction of acute photodamage in skin by topical application of a novel PARP inhibitor. Biochem. Pharmacol..

[21] Racz I., Tory K., Gallyas F., Berente Z., Osz E., Jaszlits L., Bernath S., Sumegi B., Rabloczky G., Literati-Nagy P. (2002). BGP-15—A novel poly(ADP-ribose) polymerase inhibitor—protects against nephrotoxicity of cisplatin without compromising its antitumor activity. Biochem. Pharmacol..

[22] Eroglu B., Kimbler D.E., Pang J., Choi J., Moskophidis D., Yanasak N., Dhandapani K.M., Mivechi N.F. (2014). Therapeutic inducers of the HSP70/HSP110 protect mice against traumatic brain injury. J. Neurochem..

[23] Salah H., Li M., Cacciani N., Gastaldello S., Ogilvie H., Akkad H., Namuduri A.V., Morbidoni V., Artemenko K.A., Balogh G. (2016). The chaperone co-inducer BGP-15 alleviates ventilation-induced diaphragm dysfunction. Sci. Transl. Med..

[24] Sumegi K., Fekete K., Antus C., Debreczeni B., Hocsak E., Gallyas F., Sumegi B., Szabo A. (2017). BGP-15 protects against oxidative stress- or lipopolysaccharide-induced mitochondrial destabilization and reduces mitochondrial production of reactive oxygen species. PLoS ONE.

[25] Polson A.G., Fuji R.N. (2012). The successes and limitations of preclinical studies in predicting the pharmacodynamics and safety of cell-surface-targeted biological agents in patients. Br. J. Pharmacol..

[26] Budzyński M.A., Crul T., Himanen S.V., Toth N., Otvos F., Sistonen L., Vigh L. (2017). Chaperone co-inducer BGP-15 inhibits histone deacetylases and enhances the heat shock response through increased chromatin accessibility. Cell Stress Chaperones..

[27] Batool T., Fang J., Jansson V., Zhao H., Gallant C., Moustakas A., Li J. (2019). Upregulated BMP-Smad signaling activity in the glucuronyl C5-epimerase knock out MEF cells. Cell Signal..

[28] Henstridge D.C., Bruce C.R., Drew B.G., Tory K., Kolonics A., Estevez E., Chung J., Watson N., Gardner T., Lee-Young R.S. (2014). Activating HSP72 in rodent skeletal muscle increases mitochondrial number and oxidative capacity and decreases insulin resistance. Diabetes.

[29] Yap A., Nishiumi S., Yoshida K.I., Ashida H. (2007). Rat L6 myotubes as an in vitro model system to study GLUT4-dependent glucose uptake stimulated by inositol derivatives. Cytotechnology.

[30] Szabo A., Sumegi K., Fekete K., Hocsak E., Debreczeni B., Setalo G., Kovacs K., Deres L., Kengyel A., Kovacs D. (2018). Activation of mitochondrial fusion provides a new treatment for mitochondria-related diseases. Biochem. Pharmacol..

[31] Gutiérrez-Ruiz M.C., Bucio L., Souza V., Gómez J.J., Campos C., Cárabez A. (1994). Expression of Some Hepatocyte-like Functional Properties of WRL-68 Cells in Culture Society. In vitro Cell Dev. Biol. Anim..

[32] McMahon D.K., Anderson P.A., Nassar R., Bunting J.B., Saba Z., Oakeley A.E., Malouf N.N. (1994). C2C12 cells: Biophysical, biochemical, and immunocytochemical properties. Am. J. Physiol..

[33] Wu J., Wang Y., Liu G., Jia Y., Yang J., Shi J., Dong J., Wei J., Liu X. (2018). Characterization of air-liquid interface culture of A549 alveolar epithelial cells. Brazilian J. Med. Biol. Res..

[34] Schneider E.H., Seifert R. (2010). Sf9 cells: A versatile model system to investigate the pharmacological properties of G protein-coupled receptors. Pharmacol. Ther..

[35] Zordoky B.N.M., El-Kadi A.O.S. (2007). H9c2 cell line is a valuable in vitro model to study the drug metabolizing enzymes in the heart. J. Pharmacol. Toxicol. Methods.

[36] Yoshioka Y., Kadoi H., Yamamuro A., Ishimaru Y., Maeda S. (2016). Noradrenaline increases intracellular glutathione in human astrocytoma U-251 MG cells by inducing glutamate-cysteine ligase protein via β3-adrenoceptor stimulation. Eur. J. Pharmacol..

[37] Gombos I., Crul T., Piotto S., Güngör B., Török Z., Balogh G., Péter M., Slotte P.J., Campana F., Pilbat A. (2011). Membrane-lipid therapy in operation: The HSP co-inducer BGP-15 activates stress signal transduction pathways by remodeling plasma membrane rafts. PLoS ONE.

[38] Sarszegi Z., Bognar E., Gaszner B., Kónyi A., Gallyas F., Sumegi B., Berente Z. (2012). BGP-15, a PARP-inhibitor, prevents imatinib-induced cardiotoxicity by activating Akt and suppressing JNK and p38 MAP kinases. Mol. Cell. Biochem..

[39] Schechter M.A., Southerland K.W., Feger B.J., Linder D., Ali A.A., Njoroge L., Milano C.A., Bowles D.E. (2014). An Isolated Working Heart System for Large Animal Models. J. Vis. Exp..

[40] Ogilvie H., Cacciani N., Akkad H., Larsson L. (2016). Targeting heat shock proteins mitigates ventilator induced diaphragm muscle dysfunction in an age-dependent manner. Front. Physiol..

[41] Taconic. https://www.taconic.com/rat-model/sprague-dawley.

[42] Oana F., Takeda H., Hayakawa K., Matsuzawa A., Akahane S., Isaji M., Akahane M. (2005). Physiological difference between obese (fa/fa) Zucker rats and lean Zucker rats concerning adiponectin. Metabolism.

[43] Animal Lab. http://animalab.eu/products/cd-1r-mouse..

[44] Chung J., Nguyen A., Henstridge D.C., Holmes A.G., Stanley Chan M.H., Mesa J.L., Lancaster G.I., Southgate R.J., Bruce C.R., Duffy S.J. (2008). HSP72 protects against obesity-induced insulin resistance. Proc. Natl. Acad. Sci. USA.

[45] Drel V.R., Mashtalir N., Ilnytska O., Shin J., Li F., Lyzogubov V.V., Obrosova I.G. (2006). The leptin-deficient (ob/ob) mouse: A new animal model of peripheral neuropathy of type 2 diabetes and obesity. Diabetes.

[46] Kennedy T.L., Swiderski K., Murphy K.T., Gehrig S.M., Curl C.L., Chandramouli C., Febbraio M.A., Delbridge L.M.D., Koopman R., Lynch G.S. (2016). BGP-15 Improves Aspects of the Dystrophic Pathology in mdx and dko Mice with Differing Efficacies in Heart and Skeletal Muscle. Am. J. Pathol..

[47] Isaac C., Wright A., Usas A., Li H., Tang Y., Mu X., Greco N., Dong Q., Vo N., Kang J. (2013). Dystrophin and utrophin ‘double knockout’ dystrophic mice exhibit a spectrum of degenerative musculoskeletal abnormalities. J. Orthop. Res..

[48] Takahashi N., Harada M., Hirota Y., Nose E., Azhary J.M.K., Koike H., Kunitomi C., Yoshino O., Izumi G., Hirata T. (2017). Activation of Endoplasmic Reticulum Stress in Granulosa Cells from Patients with Polycystic Ovary Syndrome Contributes to Ovarian Fibrosis. Sci. Rep..

[49] Sorensen J.C., Petersen A.C., Timpani C.A., Campelj D.G., Cook J., Trewin A.J., Stojanovska V., Stewart M., Hayes A., Rybalka E. (2017). BGP-15 protects against oxaliplatin-induced skeletal myopathy and mitochondrial reactive oxygen species production in mice. Front. Pharmacol..

[50] McQuade R.M., Stojanovska V., Stavely R., Timpani C., Petersen A.C., Abalo R., Bornstein J.C., Rybalka E., Nurgali K. (2018). Oxaliplatin-induced enteric neuronal loss and intestinal dysfunction is prevented by co-treatment with BGP-15. Br. J. Pharmacol..

[51] Faanes R.B., Merluzzi V.J., Williams N.H., Tarnowski G.S. (1979). Matching of chemotherapy to mouse strain and lymphoid tumor type to prevent tumor-induced suppression of specific T- and B-cell functions. Cancer Res..

[52] Charles River. https://www.criver.com/products-services/find-model/nmri-mouse?region=3631.

[53] Nascimento T.L., Silva M.T., Miyabara E.H. (2018). BGP-15 improves contractile function of regenerating soleus muscle. J. Muscle Res. Cell Motil..

[54] The Jackson Laboratory. https://www.jax.org/strain/100007.

[55] Wu L.L., Russel D.L., Wong S.L., Chen M., Tsai T., St. John J.C., Norman R.J., Febbraio M.A., Carrol J., Robker R.L. (2015). Mitochondrial dysfunction in oocytes of obese mothers: Transmission to offspring and reversal by pharmacological endoplasmic reticulum stress inhibitors. Developement.

[56] Xing B., Wang L., Li Q., Cao Y., Dong X., Liang Y., Wu X. (2015). Hsp70 plays an important role in high-fat diet induced gestational hyperglycemia in mice. J. Physiol. Biochem..

[57] Zhang D., Ke L., Mackovicova K., Johannes J.L., Van der Want J.J., Sibon O.C.M., Tanguay R.M., Morrow G., Henning R.H., Kampinga H.H. (2011). Effects of different small HSPB members on contractile dysfunction and structural changes in a Drosophila melanogaster model for Atrial Fibrillation. J. Mol. Cell. Cardiol..

[58] Literáti-Nagy B., Péterfai É., Kulcsár E., Literáti-Nagy Z., Buday B., Tory K., Mandl J., Sümegi B., Fleming A., Roth J. (2010). Beneficial effect of the insulin sensitizer (HSP inducer) BGP-15 on olanzapine-induced metabolic disorders. Brain Res. Bull..

[59] Literáti-Nagy Z., Tory K., Literáti-Nagy B., Kolonics A., Török Z., Gombos I., Balogh G., Vígh L., Horváth I., Mandl J. (2016). The HSP co-inducer BGP-15 can prevent the metabolic side effects of the atypical antipsychotics. Cell Stress Soc. Int..

[60] Literati-Nagy Z., Tory K., Literati-Nagy B., Kolonics A., Vígh L., Vígh L., Mandl J., Szilvássy Z. (2012). A novel insulin sensitizer drug candidate-BGP-15-can prevent metabolic side effects of atypical antipsychotics. Pathol. Oncol. Res..

[61] Cacciani N., Salah H., Li M., Akkad H., Backeus A., Hedstrom Y., Jena B.P., Bergquist J., Larsson L. (2019). Chaperone co-inducer BGP-15 mitigates early contractile dysfunction of the soleus muscle in a rat ICU model. Acta Physiol..

[62] Cappato R., Castelvecchio S., Ricci C., Bianco E., Vitali-Serdoz L., Gnecchi-Ruscone T., Pittalis M., De Ambroggi L., Baruscotti M., Gaeta M. (2012). Clinical Efficacy of Ivabradine in Patients with Inappropriate Sinus Tachycardia. J. Am. Coll Cardiol..

