# Supplementary material for: Pharmacological Overview of the BGP-15 Chemical Agent as a New Drug Candidate for the Treatment of Symptoms of Metabolic Syndrome
Source: Molecules. 2020 Jan 20;25(2):429. doi: 10.3390/molecules25020429 (PMC7024383; doi:10.3390/molecules25020429)
Supplement: Supplementary file 1 [file molecules-25-00429-s001.pdf]

Supplementary Materials

Table S1. Summary of the investigations related to the cardiovascular effects of BGP-15.

Main Effect	Aim of the Study	Methods	Dose
BGP-15 was able to reconstruct the diastolic dysfunction, and elevate the level of phosphorylation of VASP and phospholamban, without a significant effect on vascular function [17].	Investigation of the effectiveness of BGP-15 in relieving cardiac dysfunction on Goto-Kakizaki rats, and comparing its effects to metformin and pioglitazone.	The experiments were carried out on Goto-Kakizaki rats<br>Determination of body weight, fasting blood glucose and plasma insulin levels<br>Echocardiography<br>Functional Vascular Assay.<br>Western blot	10 mg/kg BGP-15, per os<br>100 mg/kg metformin, per os
BGP-15 was able to ameliorate cardiac function and reduce arrhythmic episodes [18].	Assessment of the therapeutic potential of BGP-15 in a mouse model which is able to develop heart failure and is susceptible to atrial fibrillation.	Electrocardiogram<br>Echocardiography<br>Histological analyses<br>Western blot analysis<br>Immunoprecipitation<br>RNA extraction and Northern blotting<br>Reverse transcription–quantitative PCR<br>Microarray analysis<br>Lipidomic analysis of atria<br>IGF1 assay	15 mg/kg BGP-15<br>50 μM BGP-15 on the cells
BGP-15 treatment resulted in endogenous HSP overexpression, and also protected against tachycardia remodeling [57].	Treatment of Drosophila melanogaster with BGP-15 in tachycardia.	The experiments were carried out on Drosophila melanogaster<br>Tachypacing of Drosophila and measurement of heart function parameters<br>Quantitative PCR<br>Western blot analysis<br>Structural evaluation by Light and Electron Microscopy<br>Calpain activity measurement	1 mM BGP-15
PARP is an important target of BGP-15, and BGP-15 decreases ROS levels and cell injury during ischemia–reperfusion in the heart by the inhibition of PARP activity [11].	Investigation of the molecular mechanism by which BGP-15 can protect the heart from ischemia–reperfusion injury in the Langendorff heart perfusion system.	Hearts from Wistar rats have been used for the experiments<br>Assay of NAD^+<br>DHR probe: for detection of ROS<br>Alkaline fluorescense analysis: determination of single-strand DNA breaks<br>ADP ribosylation assay<br>Assay to Test Inhibitory Effects on PARP Enzyme In Vitro<br>Lipid Peroxidation<br>Myocardial Enzyme Leakage	The medium contained 40 mg/L of BFP-15.

The oxidative damage in the heart, caused by imatinib, could be reversed by the administration of BGP-15 [38].	To identify the mechanisms by which imatinib induces cardiotoxicity and to determine how the PARP inhibitor BGP-15 can modify its progress.	The experiments were carried out by using hearts of male Wistar rats and the Langendorff rat heart perfusion system<br>NMR spectroscopy<br>Lipid peroxidation<br>Protein oxidation<br>Western blot analysis	200 μM BGP-15
PARP inhibitors reduced the ischemia-reperfusion-caused mitochondrial ROS production and oxidative damage [16].	Investigation of the effect of BGP-15 and other PARP inhibitors on oxidative cell damage caused by either ischemia-reperfusion or hydrogen peroxide.	The experiments were carried out by using hearts of male Wistar rats<br>and the Langendorff rat heart perfusion system<br>Assay of NAD+<br>Determination of DNA single-strand breaks<br>Lipid peroxidation<br>Determination of protein carbonyl content<br>Isolation of mitochondria<br>Measurement of mitochondrial enzyme activity<br>Determination of mitochondrial ROS production<br>NMR spectroscopy	40 mg/L BGP-15

Table S2. Summary of the investigations related to the effect of BGP-15 in the therapy of DMD.

Main Effect	Aim of the Study	Methods	Dose
Treatment of dystrophic mice with BGP-15, a pharmacological co-inducer of HSP 72, improved the pathology of DMD and extended the lifetime [15].	To test whether overexpression of Hsp72 in dystrophic muscles would ameliorate SERCA function.	Muscle functional analysis<br>Morphological analysis<br>SERCA activity assay<br>Alizarin red staining in skeletal preparations<br>Creatine kinase analysis<br>Real-time PCR<br>Western blot analysis<br>Relative SR Ca^{2+} sensitivity<br>SR Ca^{2+} leak<br>Caffeine-induced force responses and SR Ca21 accumulation<br>Single muscle fibre analysis<br>Wire hang test<br>Evans blue dye uptake<br>Peroxynitrite-mediated SERCA inhibition assay<br>Enriched SR isolation<br>The experiments were carried out on non-dystrophic (WT), dystrophic (mdx) and also dystrophic (dko) mice, which is the most phenotypically accurate murine model of DMD	15 mg/kg per day, oral gavage

BGP-15 exhibits a beneficial cardiac effect by improving cardiac pathology in DMD. Although BGP-15 ameliorated some aspects of dystrophic pathology, it was not able to improve skeletal muscle function in older mdx or dko mice [46].	Investigation into whether treatment with BGP-15 was beneficial in older mdx and dko mice when advanced pathology has already occurred.<br>Whether BGP-15 can preserve SERCA activity in the heart was also tested.	The experiments were carried out on mdx and dko mice<br>Assessment of skeletal muscle contractile properties<br>Echocardiographic analysis of cardiac structure and function<br>Evans blue dye uptake and histology<br>Western Blot analysis<br>SERCA activity assay<br>Superoxide indicator dihydroethidium intensity	15 mg/kg BGP-15

Table S3. Summary of the investigations related to the chemo and cytoprotective effects of BGP-15.

Main Effect	Aim of the Study	Methods	Dose
BGP-15 is a promising chemoprotective drug that can reduce the toxic side effects of taxol and cisplatin without compromising their antitumor effect [19].	To investigate the cytoprotective effect of BGP-15 on taxol and cisplatin-induced rats.	The experiments were carried out on Wistar rats<br>Electrophysiological testing	50, 100, 200 mg/kg of BGP-15, per os, once daily through the experiment<br>1.5 mg/kg cisplatin, intraperitoneally, once daily, for 5 days<br>5 mg/kg taxol, intraperitoneally, every other day for 10 days
The oxidative damage in the heart, caused by imatinib, could be reversed by the administration of BGP-15 [38].	To identify the mechanisms by which imatinib induces cardiotoxicity and to determine how the PARP inhibitor BGP-15 can modify its progress.	The experiments were carried out by using hearts of male Wistar rats and the Langendorff rat heart perfusion system<br>NMR spectroscopy<br>Lipid peroxidation<br>Protein oxidation<br>Western blot analysis	200 μM BGP-15
BGP-15 treatment prevented or markedly inhibited the development of acute renal failure caused by cisplatin treatment [21].	Investigation of the effects of BGP-15 on antitumor activity and nephrotoxicity of cisplatin.	MRI analysis<br>Evaluation of the poly (ADP-ribose) content and energy metabolism in the kidney<br>NMR spectroscopy<br>Determination of renal glutathione, superoxide dismutase, catalase content<br>Protein assay<br>Evaluation of the Bcl-x(L) content of the kidney<br>Evaluation of the ROS production in rat mitochondria<br>In vitro cytotoxicity assay<br>Transplantable mouse tumors	100, 200 mg/kg BGP-15 shortly before cisplatin treatment

BGP-15 was able to improve oxidative stress and also increased enteric neuronal survival. It alleviated oxaliplatin-induced intestinal dysfunction, thus BGP-15 could relieve gastrointestinal side-effects of chemotherapy [50].	Investigation into whether BGP-15 is able to alleviate intestinal dysfunction and oxaliplatin-induced enteric neuropathy.	The experiment was carried out on Balb/c mice<br>Assessment of mitochondrial **s**uperoxide production<br>Mitochondrial membrane potential assay<br>Histology<br>Immunohistochemistry<br>X-ray (to study gastrointestinal transit)<br>Determination of faecal water content and colonic faecal content<br>Assessment of ex vivo motility	15 mg/kg BGP-15<br>3 mg/kg oxaliplatin
BGP-15 therapy completely or partially protects skeletal muscle against deleterious side effects caused by oxaliplatin therapy [49].	Investigation of the effects of oxaliplatin therapy in mice on the mitochondria and skeletal system, and the capacity for BGP-15 to alleviate any pathological side-effects provoked by oxaliplatin.	Body composition analysis<br>Metabolism, voluntary exercise capacity and behavioral analysis<br>histology (Haematoxylin & Eosin staining, Oil Red O, Alizarin Red and Gomori Trichrome staining, Succinate Dehydrogenase)<br>Platinum detection in subcellular fractions<br>Atomic absorption spectrophotometry<br>Isolation of Flexor Digitorum Brevis (FDB) fibers<br>Determination of mitochondrial viability<br>Determination of mitochondrial superoxide production<br>Muscle protein extraction and Western blotting	15 mg/kg BGP-15<br>3 mg/kg oxaliplatin

Table S4. Summary of the investigation related to the effect of BGP-15 in paracetamol-induced cell death.

Main Effect	Aim of the Study	Methods	Dose
BGP-15 prevented translocation of AIF (apoptosis inducing factor) and mitochondrial depolarization. BGP-15 treatment attenuated the degree of acetaminophen provoked cell death [1].	Investigation of the effects of BGP-15 in acetaminophen provoked hepatocellular injury.	Examination of serum samples: aspartate aminotransferase (AST) and alanine aminotransferase (ALT)<br>Western blot analysis<br>Determination of glutathione content<br>Determination of thiol-disulfide redox state (AMS labeling)<br>RNA isolation and real-time RT-PCR	10, 20, 100, 200 mg/kg BGP-15<br>450 mg/kg acetaminophen

		Apoptosis and necrosis detection (staining with hematoxylin and eosin)<br>Immunohistochemistry of apoptosis-inducing factor<br>Bio-Rad Protein Assay	

Table S5. Summary of the investigations related to the insulin sensitizing effect of BGP-15.

Main Effect	Aim of the Study	Methods	Dose
BGP-15 markedly decreased olanzapine-induced insulin resistance [58].	To investigate the effect of BGP-15 in the treatment of olanzapine-caused metabolic side effects.	Randomized, double blind, parallel group, placebo-controlled single center study<br>In heathy volunteers<br>Intravenous glucose tolerance tests<br>Hyperinsulinemic-euglycemic clamps<br>Determination of body composition<br>Laboratory measurements (glucose, insulin, lipid, adiponectin and leptin determination)	400 mg of BGP-15 or placebo for 17 days<br>5 mg of olanzapine for 3 days and 10 mg for 14 days
Both the 200 and 400 mg BGP-15 dose groups showed markedly increased insulin sensitivity [3].	To quantify and demonstrate the insulin-sensitizing effect of BGP-15.	Multiple-dose, randomized, double-blind, placebo-controlled single center study<br>Untreated, non-diabetic, insulin resistant patients<br>Intravenous glucose tolerance tests<br>Hyperinsulinemic-euglycemic clamps<br>Determination of body composition<br>Laboratory measurements (glucose, insulin, NEFA, and glycerol adiponectin leptin, and resistin)<br>Homeostasis model assessment beta cell function<br>Homeostasis model assessment insulin resistance	200 mg or 400 mg of BGP-15, or placebo once daily, for 4 weeks
- As an insulin sensitizer, BGP-15 produced better results than metformin and equaled the effect of rosiglitazone. It also proved to be efficient in increasing insulin sensitivity in combination with a sulfonylurea	The evaluation of the insulin-sensitizing effect of BGP-15 alone and in combination with metformin, rosiglitazone, and glibenclamide. The	Insulin sensitivity was evaluated by hyperinsulinemic euglycemic glucose (HEGC) clamp prior to and at the end of the	5, 10, 20, 30, or 50 mg/kg of BGP-15 per os<br>2 mg/kg rosiglitazone per os

agent (glibenclamide). Treatment with different doses of BGP-15 showed the insulin sensitizing effect in cholesterol-fed rabbits, but not in normal rabbits. These experiments confirmed that BGP-15 has a beneficial effect only in the insulin-resistant state. The administration of BGP-15 was protective against streptozotocin-induced changes in vasorelaxation, which is similar to the effect of rosiglitazone [14].	investigation of the therapeutic effect of BGP15 and rosiglitazone on vasorelaxation.	treatment period, 4–6 hr after the last dose. The experiments were carried out on different animal models: New Zealand rabbits, Wistar and Goto Kakizaki (GK) rats, Sprague–Dawley rats.	100 mg/kg metformin per os 1 mg/kg glibenclamide per os 50 mg/kg streptozotocin i.v.
BGP-15 abolished the development of olanzapine-provoked insulin resistance Conventional insulin sensitizers, metformin and rosiglitazone in the applied doses were ineffective [59].	To compare the effect of BGP-15 with other oral antidiabetics in connection with the metabolic side effects of AAPDs.	The experiments were carried out on Wistar rats Hyperinsulinaemic Euglycaemic Glucose Clamp (HEGC)	0.005 mg/kg risperidone for 21 days, subcutaneously 20 mg/kg BGP-15 for 21 days, orally 10 mg/kg clozapine for 2 months 20 mg/kg BGP-15 for 2 months 1 mg/kg olanzapine for 28 days 10 mg/kg BGP-15 for 28 days 100 mg/kg metformin for 28 days 3 mg/kg rosiglitazone for 28 days
BGP-15 potentiates the insulin sensitizing effect of rimonabant; this occurs at much lower doses, than expected if the two drugs were administered alone [4].	To investigate the insulin sensitizing effect of BGP-15 in combination with rimonabant, another insulin sensitizing drug.	The experiments were carried out on Zucker obese rats Hyperinsulinaemic Euglycaemic Glucose Clamp (HEGC)	10 mg/kg rimonabant 30 mg/kg rimonabant 3 mg/kg BGP-15 10 mg/kg BGP-15 for 5 days

Table S6. Summary of the investigations related to the effects of BGP-15 in skin injury, TBI, and VIDD.

Main Effect	Aim of the Study	Methods	Dose
BGP-15 treatment markedly decreased the number of sunburn cells in UV radiation exposed skin; BGP-15 has a DNA protective effect if applied topically [20].	Investigation of the protective effect of BGP-15 against injuries caused by UV rays.	The experiments were carried out on hairless mice (VAF/plus CRL: hr/hr BR) Phototesting of mouse skin (determination of MED dose) Treatment with BGP-15M cream, or just its vehicle	5–20% concentration of BGP-15 in the cream

		Clinical investigations<br>Histological investigations<br>Detection of sunburn cells<br>Determination of skin absorption of BGP-15 (by using ^{14}C labeled BGP-15)<br>Determination of single-strand DNA breaks<br>ADP ribosylation assay<br>Detection of ADP ribosylation by immunohistochemical analysis<br>Detection of UV induced immunosuppression by immunostaining of TNFα and IL-10	
Hsp70/Hsp110 have a significant role in neuronal survival after TBI, and the inducers of these heat shock proteins have beneficial effects in the reduction of the pathological consequences of TBI [22].	Examination into whether those drugs that increase Hsp70/Hsp110 levels are able to protect cells against traumatic brain injury (TBI); mice were subjected to TBI and then<br>BGP-15 or Celastrol were applied.	The experiments were carried out on wild type and Hsp 70/Hsp 110 deficient mice<br>Magnetic resonance imaging<br>Immunoblotting<br>Histology and immunohistochemistry<br>Microarray analysis<br>qRT-PCR<br>Neurological injury determination	15 mg/kg BGP-15, per os<br>1 mg/kg Celastrol, intraperitoneally
BGP-15 increased diaphragm muscle fiber force generation capacity, thus decreasing the negative effects of mechanical ventilation on diaphragm muscle function [23].	Assessment of the therapeutic effect of BGP-15 on ventilation-induced diaphragm dysfunction (VIDD).	Membrane permeabilization and single muscle fiber contractility measurements<br>Modified single fiber myosin in vitro motility assay for<br>speed and force measurement<br>Transmission electron microscopy<br>Lipidomics and mass spectrometry<br>Proteomics and mass spectrometry<br>Respiratory chain enzymatic activity<br>In vitro SUMO2 conjugation assay<br>Total RNA isolation<br>qPCR<br>Immunoblotting<br>Isolation of SUMOylated proteins	40 mg/kg BGP-15 iv.

Table S7. Summary of the investigations related to effects of BGP-15 in gynecological diseases.

Main Effect	Aim of the Study	Methods	Dose

BGP-15 reduced interstitial fibrosis and collagen deposition in the ovaries [48].	Investigation the effects of BGP-15 in the treatment of PCOS mice.	The experiment was carried out on a PCOS mouse model<br>TGF-β1 assay<br>Testosterone assay<br>Immunohistochemistry<br>In situ hybridization<br>Histology<br>RNA extraction<br>RT and quantitative real-time PCR	3 mg/100g of body weight (BGP-15)
BGP-15 was able to restore mitochondrial activity in oocytes from obese mice, and it normalized fetal mtDNA transmission and embryo development [55].	Testing the ability of BGP-15 in terms of whether it reverses effects of obesity, and if it is able to restore embryo development.	Metabolite and endocrine measurements<br>RNA isolation<br>Real-time reverse transcription (RT)-PCR<br>Lipid droplet staining<br>Immunocytochemistry<br>Analysis of oocyte mitochondrial membrane potential and autophagy<br>Quantification of mtDNA copy number and sequence variants	100 mg/kg BGP-15
Administration of BGP-15 significantly reduced abnormal weight gain after pregnancy [56].	Assessment of the changes in glucose and lipid metabolisms levels after activation of Hsp70 with BGP-15.	ELISA<br>Determination of glucose, total cholesterol and triglyceride levels from blood samples<br>BAT (brown adipose tissue) thermal imaging and analysis<br>Immunohistochemistry	100 mg/kg BGP-15
BGP-15 can be a new mitochondrial drug candidate for preventing ROS-related and inflammatory disease progression [24].	Investigation into whether the protective effects of BGP-15 are in connection with reducing mitochondrial ROS production and preserving mitochondrial integrity.	HPLC-MS/MS analysis<br>Cell viability assay<br>Determination of reactive oxygen species in cell culture<br>Determination of mitochondrial production of reactive oxygen species<br>Construction of mitochondria directed enhanced red fluorescent protein<br>JC-1 assay for fluorescent microscopy<br>Tetramethylrhodamine methyl ester (TMRM) assay<br>Identification of the type of cell death by annexin V/PI staining	50 μM BGP-15
